# Using temperament and character dimensions (TCI) to analyze the personality profiles of adults and older adults with cancer managed in outpatient settings

**DOI:** 10.3389/fpsyg.2023.1289093

**Published:** 2024-01-15

**Authors:** Anna Vespa, Maria Velia Giulietti, Paolo Fabbietti, Mirko Di Rosa, Pisana Gattafoni, Rossana Berardi, Giorgio Arnaldi, Giancarlo Balercia, Roberta Spatuzzi

**Affiliations:** ^1^Scientific and Technological Area, INRCA-IRCCS National Institute of Health and Science on Aging, Ancona, Italy; ^2^Biostatistical Center, INRCA-IRCCS National Institute of Science and Health on Aging, Ancona, Italy; ^3^Scientific Direction, INRCA-IRCCS National Institute of Science and Health on Aging, Ancona, Italy; ^4^Clinic of Internal Medicine and Geriatrics, INRCA-IRCCS National Institute of Health and Science on Aging, Ancona, Italy; ^5^Department of Oncology, Ospedali Riuniti, Polytechnic University of Marche, Ancona, Italy; ^6^Division of Endocrinology, Department of Clinical and Molecular Sciences, Polytechnic University of Marche, Ancona, Italy; ^7^Department of Mental Health, ASP Basilicata, Potenza, Italy

**Keywords:** personality, temperament, character, adults, older adults, cancer

## Abstract

**Introduction:**

This study aimed to investigate profiles of personality evaluated by temperament and character dimensions (TCI) in 638 adult and older adult patients (CP) who had recently been diagnosed with breast, colon, lung, and other kinds of cancer (female and male subjects were assessed). Tests: Temperament and Character Inventory (TCI). Statistical analysis: cluster K-means analysis for personality traits.

**Results:**

Two different personality profiles emerged: “Low self-determination and pessimism” (Profile 1) and “Self-determination and self-caring (medium)” (Profile 2). The following significant differences were observed in the TCI dimensions between the two profiles: Temperament-Novelty-Seeking (NS) (*p* < 0.001); Harm-Avoidance (HA) (*p* < 0.001); Reward-Dependence (RD) (*p* < 0.001); Persistence (PS) (*p* < 0.001); Character-Self-Directness (SD) (*p* < 0.001); Cooperativeness (C) (*p* > 0.001); Self-Transcendence (ST) (*p* < 0.001). No differences in the two profiles were found between adult and elderly patients. Profile 1 - “Low self-determination and pessimism”: Patients with this profile present low resistance to frustration, poor search for novelty and solutions (NS), anxiety and pessimism (medium HA), high social attachment and dependence on the approval of others (medium-high RD), and low self-determination (PS) as temperament dimensions; and medium-low self-direction, low autonomy and ability to adapt (SD-medium-low), medium cooperativeness (C), and low self-transcendence (ST) as character dimensions. Profile 2 - “Self-determination and self-caring (medium)”: Patients with this profile have resistance to frustration, ability to search for novelty and solutions (medium-NS), low anxiety and pessimism (HA), low social attachment and dependence on approval (medium-low-RD), and determination (medium-high PS) as dimensions of temperament; and autonomy and capacity for adaptation and self-direction (SD), capacity for cooperation (high-CO), and self-transcendence (medium-high-ST) as character dimensions.

**Conclusion:**

Personality screening allows a better understanding of the difficulties of the individual patient and the planning of targeted psychotherapeutic interventions that promote quality of life and good adaptation to the disease course.

## 1 Introduction

Personality characteristics affect the long-term wellbeing and health-related quality of life (HrQoL) of cancer patients. Personality refers to the set of interconnected behavioral, cognitive, and emotional patterns of a person or individual that interact with biological and environmental factors (Koole et al., 2019). It is known that personality plays an important role in the etiology of psychosomatic diseases (Dahl, 2010; Jokela et al., 2014; Bovero et al., 2021; Lemaitre et al., 2021). Patients with some psychosomatic diseases, including cancer, present unique personality traits, such as an inability to express emotions (and/or emotional repression, particularly anger), conflict avoidance, need for approval from others, low self-affirmation, and a variety of behaviors (e.g., submissiveness, excessive patience, pathological kindness and agreeableness, cooperation), all described as Type C personalities (Blatnı and Adam, 2008; Lin et al., 2012; Batıoğlu-Karaaltın et al., 2017; Rochefort et al., 2019; Cerezo et al., 2020; Knefel et al., 2023). One of the most important common findings reported by studies is loss avoidance behavior (Rademaker et al., 2009; Aukst Margetić et al., 2013; Honorato et al., 2017; Rochefort et al., 2019; Margetić et al., 2020; Choi et al., 2022; Vespa et al., 2022). Furthermore, for some authors, personality determines whether or not the patient adapts to the disease condition, treatment (e.g., chemotherapy, cobalt therapy), and outcome (Naylor et al., 2017; Burnos and Bargiel-Matusiewicz, 2018; Margetić et al., 2020). Based on these premises, the psychobiological model of personality (TCI) developed by Cloninger et al. (1993) was considered the most appropriate for our analysis. It is based on four temperamental and three character dimensions: Harm Avoidance (HA), Novelty Seeking (NS), Reward Dependence (RD), and Persistence (P) as temperament dimensions, and Self-Directedness (SD), Cooperativeness (C), and Self-Transcendence (ST) as character dimensions (Cloninger, 1986). The temperament dimensions of personality concern the regulation of emotional reactions and habits and are predominantly based on biology, while the development of character dimensions is linked to interaction with the environment (Cloninger et al., 1993). Some studies have shown that high Harm Avoidance and low Self-Directedness have strong associations with depression in the general population (Pilevarzadeh et al., 2019; Uygur et al., 2022), as well as in different clinical populations (Ristvedt and Trinkaus, 2009; Ozcan Dag et al., 2017; Aho et al., 2022, 2023).

Therefore, TCI could be used in screening and psychotherapeutic treatment planning for cancer patients in a depressed state. Some interventions have been proven to be effective in modifying some dimensions, such as self-direction and harm avoidance (Unseld et al., 2018; Öztürk et al., 2019). High harm avoidance reflects anxious subjects who are prone to depression and pessimism, while high self-directness reflects personality features such as responsibility, determination, resourcefulness, and self-acceptance (Rademaker et al., 2009; Svrakic and Cloninger, 2010). Furthermore, differences in psychobiological model dimensions have been associated with adaptation or maladaptation to several chronic diseases, in addition to pain perception, which is a common problem in cancer patients (Hamer et al., 1999; Margetić et al., 2020).

In the present study, we can hypothesize that adults and elderly cancer patients may have different aspects and intensities of the temperament and character dimensions of personality through a cluster analysis. The short version of the TCI-R, the TCI-140, with 140 instead of 240 items, chosen for the present analysis, was developed specifically for individuals such as adults and older adults with cancer, who are often frail, with declining health conditions and multiple symptoms, and who may show fatigue and difficulty maintaining attention for long periods of time. The results of the present study may provide indications for screening and psychotherapeutic and multidisciplinary interventions aimed at addressing specific personality problems in order to help the patient cope better with the disease.

## 2 Materials and methods

### 2.1 Participants

Participants were selected from the outpatient settings of the oncology clinics of different hospitals in Ancona, Italy, namely the National Institute of Health and Science on Aging (INRCA-IRCCS) and the Polytechnic University of Marche. The protocol was approved by the Bioethics Advisory Committees of the IRCCS-INRCA (approval number: code no. 18019) and the Polytechnic University of Marche (approval number: CdB: SC/10/271/bis).

The exclusion criteria were: age <40 years; (1) symptoms or signs of cognitive impairment as assessed by the MMSE; (2) psychiatric disorders (such as borderline), as assessed using the SASB questionnaire by Benjamin et al. (2006), which describes the psychological processes of the personality structure at an intrapsychic level and is validated on the basis of the DSMIV; (3) severe symptoms of discomfort caused by cancer treatment; (4) inability to read, write, and speak Italian; and (5) inability to provide informed consent due to medical conditions.

A total of seven hundred and seventy-five participants who met all the inclusion criteria were contacted in an outpatient setting by physicians who explained the study and invited them to participate in the study project.

Seven hundred fifteen of them agreed to participate in the study and signed written informed consent. Patients completed the questionnaire either in the clinic or at home. Patients who chose to fill out the forms at home were given a stamped, self-addressed envelope to return the form. Seventy-seven patients did not complete the questionnaire and/or suffered from psychiatric disorders; therefore, it was decided not to consider them in the analysis. Thus, six hundred thirty-eight adult and older adult oncology outpatients were included in all statistical analyses. The TCI-140 was administered to all participants.

### 2.2 Measures

The Temperament and Character Inventory-140 (TCI-140) is the short version of the 240-item TCI-R (Cloninger et al., 1993; Hansenne et al., 2005; Vespa et al., 2015) and includes only the first 140 items of the TCI-R, which are rated on a five-point Likert scale (1 = definitely false; 2 = mostly or probably false; 3 = neither true nor false or about equally true and false; 4 = mostly or probably true; 5 = definitely true). Consistent with the TCI-R scoring, the TCI-140 provides a value for each major temperament dimension: Novelty Seeking (NS), Harm Avoidance (HA), Reward Dependence (RD), and Persistence (PS); character dimensions: Self-Directedness (SD), Cooperativeness (C), and Self-Transcendence (ST) of the TCI-R, as well as separate scores for each facet. For this study, the Italian version of the TCI-140 was used (Vespa et al., 2015).

The Mini-Mental State Examination (MMSE) (Folstein et al., 1975) is the most commonly used screening tool for dementia. The MMSE consists of 30 questions and includes tests of orientation, concentration, attention, verbal memory, naming, and visuospatial ability. The scores for the items are summed together into a total score. Higher scores indicate better cognition, and the maximum score is 30. The total score, which is the sum of the patient’s scores for each item, can range from a minimum of 0 (maximum cognitive impairment) to a maximum of 30 (no cognitive impairment). The cutoff score is 23–24, and most non-demented seniors rarely score below that.

### 2.3 Statistical analysis

The characteristics of the study sample are shown by the mean ± standard deviation for continuous variables or as percentages for categorical ones.

To determine two final profiles based on seven TCI subscales, a cluster k-means analysis was performed. A “Low self-determination and pessimism” profile and a “Self-determined and self-caring (medium)” profile were built.

K-means analysis requires little time to calculate distances between data points and centroids.

The algorithm is iterative, meaning that some of its phases are repeated. It consists of three steps:

- Initialization: the input parameters are defined;
- cluster assignment (centroid): each point is assigned to the closest cluster;
- update of the centroid position: recalculates the exact point of the centroid and modifies its position.

After matching the type of cancer (breast, colon, or other) with the two profiles, statistical significance was set at *p* < 0.05. All statistical analyses were performed with SPSS version 24.0 (SPSS Inc., Chicago, IL, USA).

## 3 Results

### 3.1 Cluster K-means analysis. TCI personality dimensions

The internal reliability of the TCI-140 in terms of Cronbach’s α showed good internal consistency dimensions (α = 0.776).

Two distinct personality profiles of cancer patients were identified based on scores on seven temperament and character dimensions derived from TCI-Intrex using K-means cluster analysis (Table 1).

**TABLE 1:** Cluster K-means analysis.

Variables	Final cluster centers of profiles		F	*p*
	**Profile 1 “Medium-Low self- determination and pessimism” *n* = 325** **(50.9%)**	**Profile 2 “Self-determination and self- caring (medium)” *n* = 313 (49.1%**		
NS	2.6	2.8	31,200	*p* < 0.001
HA	3.2	2.7	146,892	*p* < 0.001
RD	3.1	3.5	77,153	*p* < 0.001
PS	3.0	3.7	252,752	*p* < 0.001
SD	3.3	3.7	38, 193	*p* < 0.001
c	3.7	3.9	26,326	*p* < 0.001
ST	2.3	3.3	432,052	*p* < 0.001

TCI profiles.

#### 3.1.1 Differences in demographic characteristics between TCI personality profiles

A total of six hundred thirty-eight cancer patients participated in the study. The mean age was 56.6 (± 13.5) years. A total of 41% of the subjects were women, and 59% were men.

There were no differences between the dimensions of the two patient profiles for socio-demographic characteristics (sex, age, marital status) (Table 2). There was a difference in the level of education.

**TABLE 2:** Sample characteristics by TCI profiles.

Variables	All (*n* = 638)	Profile 1 “Medium-Low self-determination and pessimism” (*n* = 325)	Profile 2 “Self- determination and self-caring (medium)” (*n* = 313)	*p*
Female, *n* (%)	258 (41.0)	138 (42.9)	121 (38.9)	0.312
Age, years	56.6 ± 13.5	56.4 ± 13.6	56.6 ± 13.5	0.861
Education, *n* (%)				0.016
Primary school	104 (18.5)	55 (19.6)	49 (17.3)	
Secondary school	290 (51.5)	151 (54.0)	139 (49.1)	
High school	112 (19.9)	40 (14.3)	72 (25.4)	
Degree	57 (10.1)	34 (12.1)	23 (8.1)	
Marital status, *n* (%)				0.259
Married/cohabiting	216 (52.8)	113 (54.9)	103 (50.7)	
Single	39 (9.5)	23 (11.2)	16 (7.9)	
Widowed	81 (19.8)	40 (19.4)	41 (20.2)	
Separated/divorced	73 (17.8)	30 (14.6)	43 (21.2)	
Type of cancer, *n* (%)				0.224
Breast	230 (36.1)	113 (35.2)	117 (37.6)	
Colon	153 (24.0)	87 (27.1)	66 (21.2)	
Others	255 (39.9)	121 (37.7)	128 (41.2)	

The majority of patients were married (52.8%), whereas the remaining percentage were single (9.5%), widowed (19.8%), or divorced/separated (17.8%). The educational level of the total sample included patients with primary school (18.5%), middle school (51.5%), high school (19.9%), and university education (10.1%). The subjects were affected by breast cancer (36.1%), colon cancer (24%), and other kinds of cancer (lung, brain, uterus, etc.) (39.9%) (Table 2).

Furthermore, there were no differences between adult and elderly patients in the TCI dimensions (Table 2).

The proportion of patients (50.9%; *n* = 325) was classified as being in Profile 1, “Low self-determination and pessimism,” while 49.1% of the sample (*n* = 313) was classified as belonging to Profile 2, “Self-determined and self-caring (medium)” (means scores of TCI dimensions: Table 1). The distribution of cancer types in patients with the two profiles was as follows: Profile 1- breast 49.1%, colon 56.9%, other types 37.7%; Profile 2–breast 50.9%, colon 43.1%, other types 51.4%.

### 3.2 TCI-temperament and character: differences between two profiles of cancer patients cluster K-means analysis

Significant differences were found when comparing patients with Profile 1 and those with Profile 2 (Table 1).

### 3.3 TCI-temperament

Significant differences were found in the following TCI dimensions:

Novelty Seeking-NS (*F* = 31.200, *p* < 0.001) and its subdimensions: Exploratory Excitability-NS1 (*p* < 0.001), Impulsiveness-NS2 (*p* < 0.001), - Disorderliness -NS4 (*p* = 0.015). No difference emerged for Extravagance- NS3 (Table 1).
Harm Avoidance-HA (*F* = 146.892, *p* < 0.001) and its subdimensions: Anticipatory Worry - HA1 (*p* < 0.001), Fear of Uncertainty- HA2 (*p* < 0.001), Shyness-HA3 (*p* < 0.001), Fatigability and Asthenia - HA4 (*p* < 0.001) (Table 1).
Reward Dependence (RD) (*F* = 77,153, *p* < 0.001) and its subdimensions: Sentimentality - RD1 (*p* < 0.001), Openness to warm communication or social sensitivity - RD2 (*p* < 0.001), Attachment - RD3 (*p* < 0.001), Dependence on approval by others - RD 4 (*p* < 0.001) (Table 1).
Persistence (PS) (*F* = 252,752, *p* < 0.001) (Table 1), and its sub-dimensions: Eagerness of effort - PS1 (*p* < 0.001), Work hardened - PS2 (*p* < 0.001), Ambitious - PS3 (*p* < 0.001), and Perfectionist - PS4 (*p* < 0.001) (Table 1).

### 3.4 TCI character

Self-Directness (SD) (*F* = 38.193, *p* < 0.001) (Table 1) and its subdimensions: Responsibility vs. Blaming - SD1 (*p* < 0.001), Purposefulness vs. Lack of Goal Direction - SD2 (*p* < 0.001), Resourcefulness vs. Inertia - SD3 (*p* < 0.001), Self-Acceptance vs. Self-Striving - SD4 (*p* < 0.001), and Congruent Second Nature vs. Incongruent Habits - SD5 (*p* < 0.001) (Table 1).

Cooperativeness (C) (*F* = 26,326, *p* < 0.001) (Table 1), and its subdimensions: Empathy vs. Social Disinterest-C2 (*p* < 0.001), and Helpfulness vs. Unhelpfulness-C3 (*p* < 0.001). No differences emerged in Compassion vs. Revengefulness-C4, Principles vs. Self-advantage-C5, Social acceptance vs. Intolerance C1 (Table 1).

Self-Transcendence (ST) (*F* = 432.052, *p* < 0.001) (Table 1), and its subdimensions: Self-Forgetful vs. Self-Conscious Experience - ST1 (*p* < 0.001), Transpersonal Identification vs. Self-Isolation - ST2 (*p* < 0.001), Spiritual Acceptance vs. Rational Materialism - ST3 (*p* < 0.001) (Table 1).

## 4 Discussion

The Cluster K-means analysis allowed us to identify two different personality traits of temperament and character in adult and elderly cancer patients in outpatient settings: Profile 1 - “Low self-determination and pessimism” and Profile 2 “Self-determination and self-care.” These are the key findings of the present study.

The personality traits of patients with Profile 1 indicate significant adaptation difficulties with poor ability to cope with the course of the disease; on the contrary, patients with Profile 2 show remarkable adaptation skills.

Discriminating between patients with ability and those with difficulty adapting to the disease conditions may help clinicians better understand and address individual patient difficulties.

There is no difference between adult and elderly patients between the two profiles.

Furthermore, the two profiles are distributed across all tumor types.

**Profile 1 – “Low self-determination and pessimism” (LSdandP)** includes the following traits: low exploratory activity in response to novel stimulation (NS), with avoidance of frustration, worry, pessimism, shyness, being fearful, and easily tired (HA); a tendency not to respond strongly to reward signals with medium-low openness to warm communication or social sensitivity; dependence on approval by others (RD); medium-low persistence as perseverance correlated with “reward addiction” (P); medium self-determination (SD); medium expression of social tolerance, empathy, and compassion (C); low self-identification as an integral part of the whole of all things (ST).

**Profile 2 - “Self-determined** **and self-caring (medium)” (SDandSC)** includes the following traits: medium exploratory activity in response to new stimuli with the ability to cope with and avoid frustration, medium concern, low pessimism (NS), low shyness, being fearful, doubtful, and easily fatigued (HA), medium openness to warm communication or social sensitivity, medium-low dependence from others’ approval (RD), medium-high persistence as perseverance correlated with “reward addiction” (P), medium-high self-determination (SD), high expression of social tolerance, empathy, and compassion in relation to self-identification as an integral part of humanity or society (C), medium self-identification as an integral part of the whole of all things (ST).

Therefore, Profile 1 - “Low self-determination and pessimism,” highlighted the presence of a high HA and a low SD, which play an important role as indicators of poor avoidance of adverse stimuli, poor response to stressful conditions, and mood decline. Furthermore, they are indicators of poor adaptation to the disease condition and medical treatment (Aukst Margetić et al., 2013; Choi et al., 2022).

Our results are in agreement with research using the Temperament and Character Inventory-Revised (TCI-R) test, which showed that some patients are typified by high Harm Avoidance (HA) and low Self-Directedness (SD) compared to a healthy control group (Honorato et al., 2017; Öztürk et al., 2019; Choi et al., 2022).

Because a high SD reflects a person’s adaptability, Profile 2 patients with this dimension are likely to have more resources to adapt and meet the challenges of their cancer journey.

In fact, higher SD scores reflect an organized person with defined personal goals, self-determination, and autonomy (Cloninger et al., 1993). These dimensions of personality are associated with higher quality of life, subjective wellbeing, and a good mood in cancer patients (Bortolato et al., 2017; Choi et al., 2022). Profile 2 patients showed medium-high values on this dimension. It means that they are able to face the challenges due to stressful events such as the onset and course of cancer, but they may need interpersonal help. These patients showed a good value in Cooperativeness (C), which expresses social tolerance, empathy, and compassion in relation to self-identification as an integral part of humanity, society, and the whole of all things, for example, the universe (Self-transcendence-ST). Therefore, Profile 2 patients with these dimensions are able to maintain good relationships and thus receive the emotional and physical help they need.

On the contrary, Profile 1 patients may have problems with interpersonal relationships. Other studies have shown that patients with lower self-directedness and/or greater persistence appear to be prone to critical behavior toward themselves and others (Lemogne et al., 2018; Cerezo et al., 2020).

Our results are supported by other studies on women with breast cancer, which show that self-criticism, associated with poor autonomy, dependence on others, and difficulty in making free choices based on one’s emotional and practical needs, constitutes a potential factor of emotional vulnerability due to psychological distress and compromised quality of life (Vespa et al., 2022).

Furthermore, self-criticism and low autonomy are linked to difficulties in taking responsibility with little awareness of the consequences of one’s actions (Koole et al., 2019; Vespa et al., 2022).

Patients with these characteristics may have issues making free choices based on both their emotional and practical needs, neglecting themselves and their needs, even in interpersonal relationships (Lemogne et al., 2018; Cerezo et al., 2020; Aho et al., 2023; Knefel et al., 2023). Based on these considerations, they run the risk of not receiving the help they need (Oh et al., 2020).

Personality screening is a means of directing increased clinical attention (e.g., group psychoeducation and/or psychotherapy) to those patients with specific problematic personality dimensions (e.g., lower self-directedness and/or greater perseverance) who are more prone to self-criticism and neglectful behavior (Hajek et al., 2020; Oh et al., 2020).

Another dimension that differs between the two profiles is Persistence, which has been shown to predict good adaptation and is defined as the ability to perform self-care behaviors despite frustration and fatigue.

The dimension of high persistence is typical of a person who is industrious, diligent, hardworking, ambitious, and has a perfectionist behavioral strategy capable of dealing with stable contingencies. On the other hand, too much persistence can be maladaptive because it causes stress in situations that change quickly and unexpectedly, such as dealing with a life-threatening illness (Cloninger et al., 1993).

Therefore, it is important to evaluate this dimension in the context of the personality in which it appears. Our results showed that the patients with Profile 1 scored lower on this dimension than those with Profile 2.

Consequently, the temperament scores in Novelty Seeking (NS) allow us to define subjects with Profile 1 as people who may show difficulty regulating emotions, are not inclined to commitment, and have a low tolerance for frustration. They may also develop inconsistent and/or superficial relationships to avoid experiencing any kind of failure.

The medium-low score on the Self-Directedness (SD) scale of Profile 1 patients indicates fragile, unstable people who do not seem to have a main internal organization, so much so that they appear to have poor integrity.

This aspect manifests itself in the inability to define, plan, and achieve goals. Finally, these are subjects who frequently change their motivations and who have short-lived emotional and life experiences, probably related to the fact that they are not able to manage certain emotions and circumstances. These issues can be considered an indication of poor adaptation to the course of the disease and medical therapies.

Therefore, these patients should be subjected to closer surveillance through personality screening aimed at planning targeted multidisciplinary and psychotherapeutic interventions (Hajek et al., 2020).

In the case of patients with Profile 2 who are in distress, a targeted intervention to support the patient, involving their family, may be sufficient.

Limitations: Our results provide a snapshot of temperament and character after cancer diagnosis and during the treatment phase. These dimensions may differ during the treatment phase or at other points in the course of cancer progression. Future longitudinal studies could make these comparisons and test differences in temperament and character at different stages of the cancer journey.

## 5 Conclusion

Our findings suggest that personality screening within standard time periods may allow the identification of patients with poor adaptation to cancer treatment (Unseld et al., 2018). Indeed, knowledge of differences in personality profiles could provide information on patients most at risk for difficulties in adapting to cancer treatment, as well as provide indications for more targeted multidisciplinary and psychotherapeutic interventions (Semenenko et al., 2023).

Based on our results, we can suggest that specific and multidisciplinary psychotherapeutic interventions such as psychodynamic psychotherapy (Luyten and Fonagy, 2020), cognitive behavioral therapy (Ardizzone et al., 2022; Lacasta and Cruzado, 2023; Torres-Blasco et al., 2023) and interpersonal reconstructive therapy (Benjamin, 2003) could be the most appropriate and effective with patients with a profile of “low self-determination and pessimism.”

We also suggest that integrated holistic psychotherapy and/or cognitive behavioral therapy with mindfulness-based interventions (MBIs) (Xunlin et al., 2020) may instead be more desirable for patients with Profile 2 (“Self-determination and caring”) to promote greater awareness and use of resources for a good quality of life.

## Data availability statement

The raw data supporting the conclusions of this article will be made available by the authors, without undue reservation.

## Ethics statement

The studies involving humans were approved by the Bioethic Committee- IRCCS-INRCA. The studies were conducted in accordance with the local legislation and institutional requirements. Written informed consent for participation in this study was provided by the participants’ legal guardians/next of kin. Written informed consent was obtained from the individual(s) for the publication of any potentially identifiable images or data included in this article.

## Author contributions

AV: Conceptualization, Data curation, Formal analysis, Methodology, Supervision, Validation, Writing – original draft, Writing – review and editing, Visualization. MG: Conceptualization, Data curation, Investigation, Methodology, Project administration, Writing – original draft, Writing – review and editing, Visualization. PF: Data curation, Formal analysis, Methodology, Software, Validation, Writing – original draft. MD: Data curation, Formal analysis, Methodology, Software, Validation, Writing – original draft. PG: Formal analysis, Investigation, Project administration, Writing – original draft, Writing – review and editing. RB: Conceptualization, Investigation, Project administration, Writing – original draft. GA: Conceptualization, Investigation, Methodology, Project administration, Supervision, Writing – original draft. GB: Conceptualization, Investigation, Project administration, Supervision, Writing – original draft. RS: Conceptualization, Methodology, Writing – original draft, Writing – review and editing.
